# Case Report: CBD Cigarettes for Harm Reduction and Adjunctive Therapy in a Patient With Schizophrenia and Substance Use Disorder

**DOI:** 10.3389/fpsyt.2021.712110

**Published:** 2021-07-22

**Authors:** Maximilian Meyer, Marc Walter, Stefan Borgwardt, Alexandra Scheidegger, Elisabeth Lang, Patrick Köck

**Affiliations:** ^1^Department of Psychiatry (Universitäre Psychiatrische Kliniken), University of Basel, Basel, Switzerland; ^2^Department of Psychiatry and Psychotherapy, University of Lübeck, Lübeck, Germany

**Keywords:** case report, substance use disorder, cannabidiol, schizophrenia, psychosis

## Abstract

The treatment of patients with schizophrenia and substance use disorder poses a challenge for clinicians. Continued use of cannabis and cocaine can exacerbate psychotic symptoms and worsen the course of disease. To date, no pharmacotherapy is available for patients with cannabis use disorder (CUD). Cannabidiol (CBD) and Δ9-tetrahydrocannabinol (THC) are the main active constituents in *Cannabis sativa*, with the latter being linked to an increased risk of psychosis. We describe a clinical case of a male patient diagnosed with schizophrenia, combined personality disorder, CUD and cocaine use disorder. Over the course of 8 years, he was hospitalized 30 times due to psychotic relapses and continued substance use. Consequently, CBD cigarettes with a low THC content (<1%) were used as adjunctive therapy. Additionally, we established off-label treatment with methylphenidate to support abstinence. The patient reported to feel significantly less need to consume illegal cannabis with a high THC content. He stopped to use cocaine, for the time being, and has not been hospitalized since. This case report demonstrates the potential of smoked CBD as a substitute for severe and chronic CUD.

## Introduction

Pharmacotherapy of cannabis use disorder (CUD) has proven to be challenging (1, 2). Nabiximols, a combination of Δ9-tetrahydrocannabinol (THC) and cannabidiol (CBD), is available as oromucosal spray (Sativex®) and has been shown to reduce withdrawal symptoms and craving in cannabis dependent patients (3, 4). However, treatment attempts for CUD with CBD alone are scarce (5). A recent double-blind, randomized controlled trial provided first evidence for the use of CBD in severe CUD (6). Oral CBD reduced cannabis use and withdrawal symptoms and was well-tolerated (6). Due to the legal status of cannabis, the accessibility of CBD products is limited in several countries. In Switzerland, cannabis with a THC content of <1% is available for purchase on the open market (7). THC and CBD are the main active constituents in *Cannabis sativa* (8). THC has been linked to an increased risk of psychosis and exacerbated symptoms of schizophrenia (9, 10). Interestingly, some studies suggest CBD as potentially antipsychotic (11). Hence, CBD cigarettes might be a potential substitute for patients with schizophrenia and comorbid CUD. To our knowledge, the impact of smoked CBD has not yet been investigated.

This report aims to present an illustrative case of a patient with cocaine and cannabis use disorder and schizophrenia and combined personality disorder, who significantly benefited from additional treatment with CBD cigarettes.

## Case Report

The patient is a 30-year-old male who was born in Turkey and is of Kurdish descent. He moved to Switzerland in 2009 at the age of 19. In 2010, he married a Swiss woman with whom he has one child. They divorced in 2016 and he has been living alone in a rented apartment since.

He first presented in our outpatient clinic at the age of 22 in late 2012. He reported that he had lost his driving license due to cocaine use while driving and that the relationship with his wife had ended. At this time, he was unemployed, smoked cannabis 2-3 times a week and used alcohol regularly. The Beck depression inventory (BDI) showed a moderate depression with a score of 26. Over the following years, he was hospitalized 30 times in our clinic, voluntarily as well as involuntarily (see Table 1). He was diagnosed with cannabis use disorder (2014), mixed personality disorder with narcissistic and antisocial traits (2015), cocaine use disorder (2016) and schizophrenia (2018) according to ICD-10.

**Table 1:** Number of admissions from 2014 to 2021.

	**2014**	**2015**	**2016**	**2017**	**2018**	**2019**	**2020**	**2021**
# of total hospitalizations	1	1	5	2	4	7	8	2
# of involuntary hospitalizations						1		2
# forensic hospitalizations		1						

He first admitted himself to our clinic in 2014. He wished to cease cannabis consumption (about five joints a day) and reported depressive symptoms. In 2015 he was incarcerated for criminal assault. During his imprisonment, he committed a suicide attempt (self-administered lacerations at his neck and sternum). Consequently, the patient was admitted to a psychiatric forensic department, where he removed a lightning bulb in the isolation room and injured himself on the left wrist. In 2016, at the age of 25, he reported to suffer from acoustic hallucinations for the first time (imperative voices telling him to consume drugs) and that he had experienced these symptoms for 4 years. It was unclear whether this first documented psychotic episode was caused by substance use alone and he did not meet the criteria for paranoid schizophrenia according to ICD-10 at the time. During treatment, he stated that the use of cannabis would positively affect his hallucinations. He also reported to gamble a lot, losing up to CHF 9,000 in a single month. Additionally, he repeatedly expressed suicidal thoughts, like the idea of ending his life with a drug overdose. From 2016 onwards, he continued to admit himself or was referred to our clinic through his practicing psychiatrist. He alternately presented psychotic and depressive symptoms, besides the wish for detoxification and withdrawal-treatment due to continued heavy cocaine (1–2 g/day), alcohol (5–8 liters beer/day), and cannabis (3–5 joints/day) use. In 2018, the psychotic symptoms persisted despite the patient's abstinence from cannabis and cocaine, hence the diagnosis of paranoid schizophrenia was made. Due to the severity of his psychotic symptoms inpatient treatments focused on psychoeducation and the establishment of pharmacotherapy with atypical antipsychotics. In between these hospitalizations, the patient repeatedly stopped his medication (see Table 2) and resumed cannabis, cocaine, and alcohol use.

**Table 2:** Prescribed medication at discharge over 8 years.

**Year**	**2014**	**2015**	**2016**					**2017**		**2018**				**2019**							**2020**								2021	
Hospital admission	1	2	3	4	5	6	7	8	9	10	11	12	13	14	15	16	17	18	19	20^*^	21	22	23	24	25	26	27	28	29^*^	30^*^
**Antidepressants (mg)**																														
Mirtazapine	45									15																				
Trazodone											100	100																		
Sertraline													200																	
**Antipsychotics (mg)**																														
Risperidone		2	3	3						4		2	1																	
Quetiapine			50	250									100	25	50													25		
Aripiprazole					10	10	10														10			10		10		5		X
Olanzapine					15	15	15	5													10	20	20						5	
Paliperidone											X	X	X	X	X	X	X	X												
Brexpiprazole																													1	
**Sedatives (mg)**																														
Lorazepam				4						4				4				1												
Oxazepam								52,5																						
Zopiclone										7,5																				
Diazepam																														5
**Anxiolytics (mg)**																														
Pregabalin																	300	300	450											
**Stimulants (mg)**																														
Methylphenidate																														100

* *Involuntary admission X = intramuscular depot medication. Gray and white shadows are differentiate the years*.

At the end of 2019, he was involuntarily committed after shouting and rampaging in public, attacking the arresting police officers, and smashing his head against the police station's wall. He was taken to the hospital, where he had to be sedated with propofol, as his head injury could not be examined otherwise. After that, he was transferred to our psychiatric clinic, where he stated that he could not remember the events that led to his committal. He reported that he had not used cocaine within the last few months but corrected himself after a toxicologic serum screening showed positive for cocaine, cannabis, and alcohol.

In May 2020, the patient wanted to admit himself to our clinic yet again. When he was told that an immediate inpatient admission was not possible at the time, he took 20 mg of lorazepam and 55 mg of aripiprazole presumably to force his admission. As sedation wore off, the patient became increasingly aggressive and eventually attacked and injured a staff member, which led to two police-assisted isolation. The next day he apologized for his actions but was subsequently banned from entering the clinic for a month for attacking and injuring a staff member.

In January 2021, he was involuntarily committed (29th hospitalization) after getting involved in a brawl. At this time, he was intoxicated, psychotic, and did not cooperate with our staff. Again, he had to be sedated with propofol as otherwise a cranial computer tomography to rule out head injuries would not have been possible. During his hospitalization, a knife with a blade length of 15 cm was found in his room by our staff. The confiscation led to aggressive behavior and threats from the patient, leading to another ban from our clinic.

In his most recent involuntary hospitalization (30th), the police reported that the patient went on a rampage, damaging cars in the city center. He was intoxicated (alcohol) but refused to give a urine sample for further toxicological examination. He was delusional and paranoid at the time of admission (fear of getting poisoned). Throughout the hospitalization, the patient started damaging the inventory of our department. An application for compulsory treatment authorization was granted as per article 434, Swiss civil code. After switching from oral olanzapine to oral aripiprazole, the patient agreed to an intramuscular depot medication with aripiprazole. However, he continued to use cocaine and cannabis during hospitalization. Therefore, after the patient gave his informed consent, methylphenidate 100 mg/d was prescribed off-label to support cocaine abstinence.

At the same time, we initiated treatment with CBD-rich cigarettes (Manufacturer: Koch & Gsell; Products: “Heimat” Pure Hemp, “Heimat” Tobacco+Hemp; approx. 10% of CBD, <1% of THC) as adjunctive inpatient therapy. A week later in the following therapeutic session, the patient stated that he could now also abstain from illicit cannabis use. He replaced his former habit of smoking cannabis joints (illicit cannabis and standard tobacco) with CBD-rich cigarettes (approx. 20 cigarettes per day, approx. 400 mg of CBD per day). Interestingly, the patient managed to reflect on his cannabis use and its negative impact on his schizophrenia for the first time. He began to realize the relation between worsening of psychotic symptoms and continued use of cannabis containing high THC contents. Additionally, he claimed that “street cannabis would destroy my therapy with aripiprazole.” He maintained cocaine and illicit cannabis abstinence until discharge from our ward. The urine test for cocaine showed a negative result at the time of discharge, 2 weeks after initiation of methylphenidate. THC showed positive as our tests were not able to distinguish between illicit cannabis and CBD-rich cigarette consumption. For the assessment of the patient's compliance regarding the cessation of illicit cannabis use, we relied on our clinical impression and self-reports.

We established a controlled daily dispense of methylphenidate and monthly intramuscular injections of aripiprazole (400 mg/28 days) via a local pharmacy. Once weekly psychotherapeutic outpatient contacts were scheduled, and he received weekly rations of CBD cigarettes. The cantonal social department approved to cover the costs of a CBD product for half a year. For financial reasons, we suggested the purchase of “Alplant Skywalker Lemon OG Outdoor, 50 grams” (CBD-rich hemp <7% CBD, <0.2% THC) which was used by the patient to roll his cigarettes. The patient receives a package of this product every week. He continues to appear regularly at the weekly outpatient appointments. Furthermore, he reported significantly reduced illegal cannabis use but admitted some relapses. When reflecting upon the cannabis use, he started to see the link between psychotic symptoms and substance use. Due to this realization, the patient now prefers CBD rather than ordinary cannabis. He refrained from using cocaine since establishing the off-label therapy with methylphenidate. A 6-week and a 12-week follow-up urine test resulted negative for cocaine. No further psychiatric hospitalization was required since discharge. Aided by our clinic's social workers, a supportive long-term therapeutic setting is now being planned. However, his psychotic symptoms (delusion and paranoia) did not completely remit.

## Discussion

SUDs are particularly common in patients with schizophrenia with a lifetime prevalence of SUDs exceeding 40% in epidemiological studies (12). Treatment outcomes are often poor in dual diagnosis patients, since comorbid cannabis or cocaine use can cause or exacerbate psychotic symptoms (9, 10). Therefore, hospitalization and readmission are frequent in these patients, and this “Revolving door” phenomenon adds another challenge to the therapeutic efforts of clinicians (13). Since crime perpetration is more common in dual-diagnosis patients (14), incarcerations may interrupt treatment continuum. Consensus exists in the literature that integrated therapy settings which combine multiple therapy arms yield better treatment outcomes than sequential or parallel treatment settings (15, 16).

This report describes a patient for whom a suitable treatment regimen could not be established for many years. The combined use of cocaine and cannabis regularly exacerbated his schizophrenia leading to voluntary and involuntary admissions. For cocaine dependence, no pharmacological substitutes have been approved to this date. However, treatment attempts with off-label methylphenidate prescriptions have been conducted with mixed results (17). The suggestion that stimulants can cause psychotic events and should therefore be avoided in patients with a history of psychosis (18) has been challenged lately. A recent study found that the use of methylphenidate does not increase the immediate risk of psychotic events in these patients and even observed a reduced long-term risk of psychotic exacerbation (19). As with cocaine, no pharmacotherapies for CUD are available. However, treatment with orally administered CBD has yielded first results in reducing withdrawal symptoms in a phase 2a trial (6).

THC contents of non-medical (illegal) retail cannabis commonly exceed 20% (20), which poses a risk of worsening the course of psychotic disorders. Therefore, therapeutic strategies for schizophrenia and comorbid CUD are needed. Second generation antipsychotics have been recommended in dual diagnosis patients, with some of them being able to reduce alcohol, cocaine and cannabis use (21). This report illustrates a successful treatment with CBD cigarettes and off-label methylphenidate in addition to antipsychotic treatment with aripiprazole. From our perspective, the use of CBD cigarettes as an alternative to ordinary cannabis enabled this patient to better understand and cope with his schizophrenia. Additionally, the patient managed to abstain from cocaine use due to simultaneous treatment with off-label methylphenidate. Therefore, the outcome of our treatment cannot be attributed exclusively to the use of CBD cigarettes. The use of methylphenidate aimed to support cocaine abstinence, whereas the implementation of CBD cigarettes aimed to facilitate cannabis abstinence. Unlike the use of methylphenidate in cocaine use disorder, the use of CBD cigarettes in CUD constitutes a novel treatment approach. The patient increasingly succeeded in leading reflective discourse about his substance use and its interplay with his mental condition, violent behavior, and effectiveness of the antipsychotic medication. A cycle of worsening psychopathological symptoms, antisocial behavior, and the “Revolving door” phenomenon ended, at least for the time being.

## Conclusions

Cannabis and cocaine use can worsen the clinical course in patients with schizophrenia and exacerbate psychotic symptoms. Patients with schizophrenia and comorbid CUD are often unable to reduce or cease their cannabis use. Replacing non-medical cannabis with products low in THC, like CBD cigarettes, might support reduced overall THC intake in these patients without them having to change their accustomed form of consumption.

## Limitations

This case report has several limitations. First, this report is retrospective. Also, no structured interviews like the Positive and Negative Syndrome Scale (PANSS) to assess psychotic symptoms during the hospitalization nor during the outpatient period were conducted. Furthermore, the period of “successful treatment” may be short compared to the long psychiatric history and substance use duration in this case. Nevertheless, we found that the patient's perspective regarding his substance use and its impact on psychotic symptoms dramatically changed, although we did not use standardized and validated questionnaires to assess craving symptoms. We emphasize that “treatment success” is not attributable to CBD cigarettes alone. Instead, this case illustrates an integrated psychiatric effort, with CBD cigarettes as a harm-reductive intervention. Lastly, as this report only involves one patient, further studies are needed to assess this novel approach in a larger population.

## Data Availability Statement

The original contributions presented in the study are included in the article/supplementary material, further inquiries can be directed to the corresponding author.

## Ethics Statement

Written informed consent was obtained from the individuals for the publication of any potentially identifiable images or data included in this article.

## Author Contributions

MM and PK conceived and designed the case report and gathered the data. MM drafted the manuscript. PK was the treating physician and reviewed and edited the manuscript. MW, SB, EL, and AS provided substantial intellectual input. All authors contributed to the article and approved the submitted version.

## Conflict of Interest

The authors declare that the research was conducted in the absence of any commercial or financial relationships that could be construed as a potential conflict of interest.
